# A Novel Visceral Adiposity Index for Prediction of Type 2 Diabetes and Pre-diabetes in Chinese adults: A 5-year prospective study

**DOI:** 10.1038/s41598-017-14251-w

**Published:** 2017-10-23

**Authors:** Jinshan Wu, Lilin Gong, Qifu Li, Jinbo Hu, Shuping Zhang, Yue Wang, Huang Zhou, Shuming Yang, Zhihong Wang

**Affiliations:** The First Affiliated Hospital of Chongqing Medical University, Department of Endocrinology, Chongqing, 400016 China

## Abstract

The Chinese visceral adiposity index (CVAI) is a recently developed indicator of visceral adiposity. We investigated the predictive value of the CVAI for the development of dysglycemia (pre-diabetes and type 2 diabetes) and compared its predictive power with that of the Visceral adiposity index (VAI) and various anthropometric indices. This community-based study included 2,383 participants. We assessed the predictive power of adiposity indices by performing univariate and multivariate binary logistic regression analysis and calculating the area under the receiver-operating characteristic (ROC) curve according to their quartiles. Logistic regression analysis showed that individuals in higher CVAI quartiles at baseline were more likely to develop dysglycemia than those in lower CVAI quartiles. The area under the ROC curve for CVAI was significantly higher than that of other adiposity indices. In addition, among the various adiposity indices tested, the CVAI had the greatest Youden index for identifying dysglycemia in both genders. Our data demonstrate that the CVAI is a better predictor of type 2 diabetes and pre-diabetes than the VAI, BMI, waist circumference, waist-to-hip ratio and waist-to-height ratio in Chinese adults.

## Introduction

Obesity, particularly visceral obesity, is associated with an increased risk of metabolic diseases, such as dysglycemia (pre-diabetes and type 2 diabetes), dyslipidemia, hypertension, and cardiovascular disease^1^. Type 2 diabetes (hereafter referred to as diabetes), which cannot be cured, is one of the most common obesity-associated complications and has become a worldwide health burden, especially in developing countries^2^. Pre-diabetes, including impaired fasting blood glucose and impaired glucose tolerance, is a warning sign of diabetes. The Centers of Disease Control and Prevention National Diabetes Statistics Report suggested that 33.9% of U.S adults older than 18 years and 48.3% of those older than 65 had pre-diabetes in 2015–2017^3^. As to the Chinese adult population, a cross-sectional survey in 2010 reported that the estimated prevalence of pre-DM was 50.1%^4^. Therefore, early and accurate identification of visceral obesity is urgently needed to prevent dysglycemia.

Magnetic resonance imaging (MRI) and computed tomography (CT) are the gold standard for determining visceral obesity. However, they are unsuitable for clinical practices because they are time-consuming, costly and harmful. Therefore, several surrogate indices have been defined, including body mass index (BMI) and waist circumference (WC), which are simple and commonly used. The prevalence of diabetes increases with increasing BMI^5^ and WC^6,7^. Nevertheless, BMI cannot distinguish between muscle and fat body mass^8^ or between peripheral fat and abdominal fat^9^, and WC is sensitive to height and weight^10^. Other surrogate indices proposed include the waist-to-hip ratio (WHR), waist-to-height ratio (WHtR), and visceral adiposity index (VAI)^11^. The VAI, which is the product of WC, BMI, serum triglycerides (TG), and high density lipoprotein-cholesterol (HDL-C) levels, was established to estimate visceral adiposity and was validated by abdominal MRI after first modeled using logistic regression. However, the accuracy of these indices controversial, and more reliable indicators are necessary.

Considering the characteristic of body fat in an Asian population compared with that in Caucasians. Xia *et al*.^12^ developed a new surrogate index, the Chinese visceral adiposity index (CVAI), using a multivariate regression model. This index was confirmed by CT and combines demographic (age), anthropometric (BMI and WC) and metabolic characteristics (TG and HDL-C). It was found to be a reliable index for the evaluation of visceral fat dysfunction in a cross-sectional study with 485 subjects conducted in China and was further validated in a study with 6,495 subjects recruited from Changfeng, Shanghai^12^. However, no prospective study has been performed to determine whether the CVAI can predict future dysglycemia. Thus, in this 5-year prospective study our primary objective was to evaluate the predictive value of the CVAI for the development of dysglycemia. The secondary objective of this study was to compare the predictive value of the CVAI with that of other obesity indices.

## Results

### Baseline characteristics according to CVAI index quartiles

The sex-specific baseline characteristics of study subjects are shown online in Supplementary Table S1. Males and females differed significantly in all baseline characteristics. Compared to females, males had a higher mean age, systolic blood pressure (SBP), diastolic blood pressure (DBP), CVAI, WC, hip circumference (HC), BMI, WHR and WHtR. In contrast, males had a lower VAI than females. In addition, males had higher concentration of fasting plasma glucose (FPG), 2 hours postload plasma glucose (2hPG) and TG, whereas females had higher concentration of total cholesterol (TC), HDL-C, and low density lipoprotein-cholesterol (LDL-C). In total, 350 of 2,383 subjects without diabetes at baseline developed diabetes (incidence 14.7%), and 942 of 2,260 subjects without dysglycemia at baseline developed pre-diabetes (incidence 41.7%) (Supplementary Table S2). The incidence of diabetes and pre-diabetes was significantly higher in males than in females. The baseline characteristics of the study population according to CVAI quartiles are shown in Table 1. As expected, age, weight and various adiposity indices increased across the CVAI quartiles. The values for FPG, 2hPG, TG, TC and LDL-C increased, and the HDL-C level decreased with increasing CVAI quartiles. The rates of current smokers, former smokers, drinkers, and overweight and obese subjects were also significantly (P < 0.001) higher among subjects in higher CVAI quartiles. Furthermore, the prevalence of diabetes and pre-diabetes increased significantly (P < 0.001) in proportion with higher CVAI levels at baseline (Supplementary Table S2).

**Table 1:** Baseline characteristics of study subjects (n = 2,383) divided according to the quartiles of CVAI.

	Baseline CVAI				
	1st <63.84	2nd 63.84–88.74	3rd 88.75–113.88	4th >113.88	*P* value
Number (men/women)	596 (203/393)	596 (326/270)	596 (378/218)	595 (381/214)	<0.001
Age, y	49.45 ± 11.68	55.56 ± 10.81	58.96 ± 11.45	63.77 ± 11.28	<0.001
Weight, kg	54.02 ± 6.28	59.91 ± 7.47	64.10 ± 8.55	69.98 ± 9.53	<0.001
WC, cm	72.42 ± 4.90	78.69 ± 4.65	83.42 ± 4.97	90.85 ± 6.22	<0.001
HC, cm	89.36 ± 4.31	92.45 ± 4.22	95.09 ± 4.04	99.81 ± 4.53	<0.001
BMI, kg/m^2^	21.18 ± 1.97	22.92 ± 1.92	24.29 ± 2.03	26.53 ± 2.16	<0.001
VAI	1.24 ± 0.69	1.71 ± 0.92	2.32 ± 1.44	2.94 ± 1.68	<0.001
WHR	0.81 ± 0.05	0.85 ± 0.04	0.88 ± 0.04	0.91 ± 0.05	<0.001
WHtR	0.45 ± 0.03	0.49 ± 0.03	0.51 ± 0.05	0.56 ± 0.04	<0.001
SBP, mmHg	110 (100,120)	120 (110,130)	120 (110,130)	128 (120,140)	<0.001
DBP, mmHg	70 (64,80)	78 (70,80)	80 (70,80)	80 (78,86)	<0.001
FPG, mmol/L	4.10 ± 0.47	4.14 ± 0.53	4.23 ± 0.58	4.41 ± 0.67	<0.001
2hPG, mmol/L	4.88 ± 0.99	5.00 ± 1.07	5.23 ± 1.23	5.71 ± 1.54	<0.001
TC, mmol/L	4.41 ± 0.67	4.67 ± 0.78	4.76 ± 0.79	4.77 ± 0.79	<0.001
TG, mmol/L	0.96 ± 0.45	1.29 ± 0.57	1.64 ± 0.79	1.91 ± 0.85	<0.001
LDL-C, mmol/L	2.65 ± 0.60	2.91 ± 0.61	3.04 ± 0.62	3.10 ± 0.65	<0.001
HDL-C, mmol/L	1.26 ± 0.26	1.17 ± 0.23	1.10 ± 0.22	1.04 ± 0.20	<0.001
Smoking, n (%)					
Current smokers	61 (10.2)	92 (15.4)	113 (19.0)	108 (18.2)	<0.001
Former smokers	5 (0.8)	33 (5.5)	38 (6.4)	55 (9.2)	<0.001
Drinking, n (%)	28 (4.7)	56 (9.4)	57 (9.6)	79 (13.3)	<0.001
Overweight, n (%)	34 (5.7)	164 (27.5)	299 (50.3)	376 (63.2)	<0.001
Obese, n (%)	2 (0.3)	2 (0.3)	27 (4.5)	149 (25.0)	<0.001

CVAI, Chinese visceral adiposity index; WC, waist circumference; HC, hip circumference; VAI, visceral adiposity index; SBP, systolic blood pressure; DBP, diastolic blood pressure; FPG, fasting plasma glucose; 2hPG, 2 hours postload plasma glucose; TC, total cholesterol; TG, triglyceride; HDL-C, high-density lipoprotein cholesterol; LDL-C, low-density lipoprotein cholesterol.

### Prediction of the development of diabetes and pre-diabetes using the CVAI and other obesity indices

**Figure 1:**
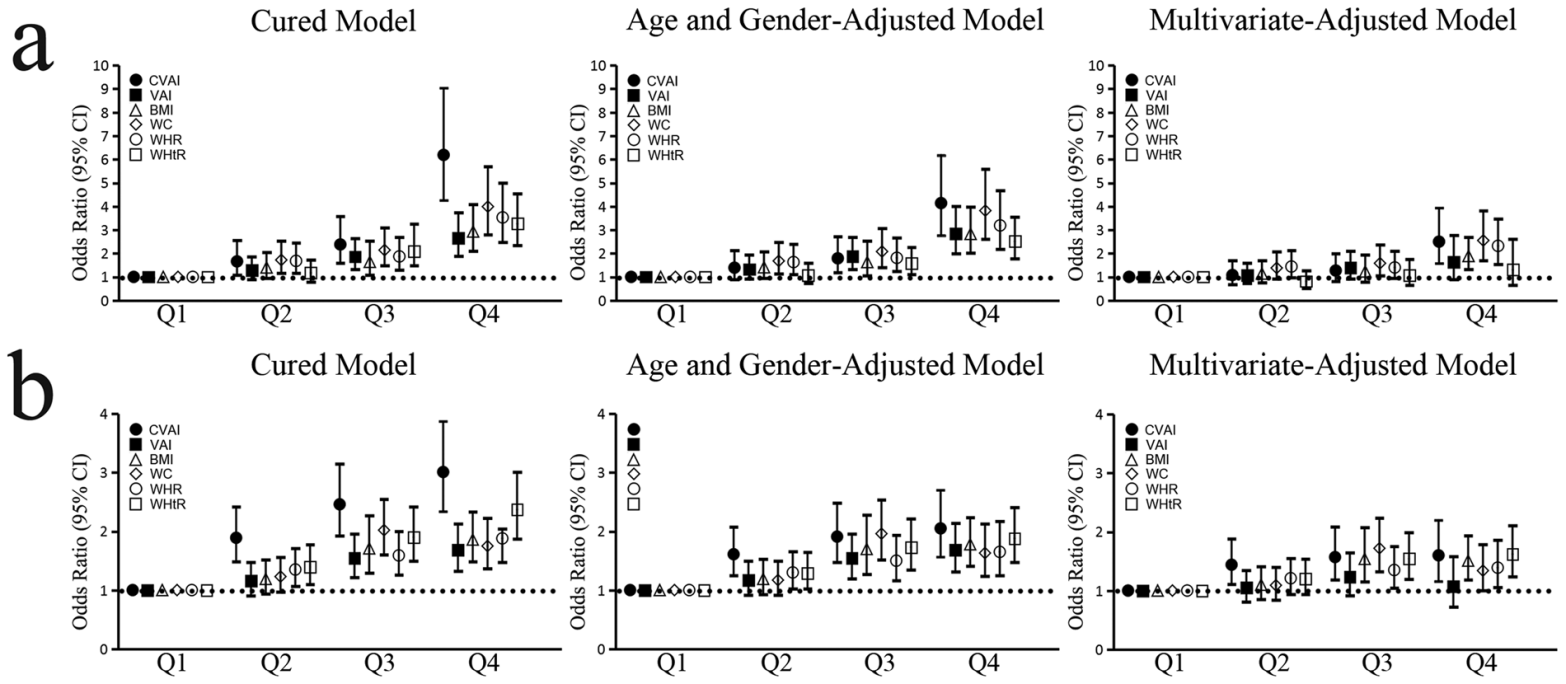
Odds ratio (OR, 95%CI) of various visceral obesity indices across their quartiles for diabetes (**a**) and pre-diabetes (**b**). Multivariate adjusted: the model that adjusted age, gender, TG, TC, LDL-C, SBP and DBP.

We used univariate and multivariate binary logistic regression to estimate the risk of dysglycemia among different CVAI quartiles. In an unadjusted model, all six obesity indices were positively associated with the incidence of diabetes (Fig. 1a). The CVAI was the strongest predictor of dysglycemia. Compared to subjects in the lowest CVAI quartile, the risk of diabetes was 6.19 (95%CI: 4.25–9.03; P < 0.001) times higher for subjects in the highest quartile; 2.38 times higher for subjects in the third quartile (95%CI: 1.58–3.57; P < 0.001) and 1.66 times higher for subjects in the second quartile (95%CI: 1.08–2.55; P = 0.023). After adjusting for age and gender as confounders, the association was reduced but remained significant. After further adjustment for potential confounders, including age, gender, TG, TC, LDL-C, SBP and DBP, the association of VAI and WHtR with diabetes decreased to nonsignificant levels. The association between the CVAI and diabetes remained significant and was the strongest among all six obesity indices. The odds ratio (OR) (2.5; 95%CI: 1.58–3.93; P < 0.001) between the lowest and the highest CVAI quartiles were still significantly different. Figure 1b displays the OR for pre-diabetes. The CVAI was the strongest predictor for pre-diabetes, followed by the WHtR and then the VAI, BMI, WC and WHtR, which showed similar predictive abilities. In an unadjusted model, compared to subjects in the lowest CVAI quartile, the risk of pre-diabetes was 3.01 (95%CI: 2.34–3.87; P < 0.001) times higher for subjects in the highest quartile, 2.46 times higher for subjects in the third quartile (95%CI: 1.93–3.15; P < 0.001) and 1.89 times higher for subjects in the second quartile (95%CI: 1.48–2.42; P < 0.001). After adjusting for age and gender, the association between the obesity indices and pre-diabetes remained significant. After further controlling for potential confounders (TG, TC, LDL-C, SBP and DBP), the association between obesity indices and pre-diabetes decreased but remained significant, with the exception of the VAI. The association between the CVAI and pre-diabetes remained significant and was the strongest among all six obesity indices. In the multivariate-adjusted model, compared to subjects in the lowest CVAI quartile, the risk of pre-diabetes was 1.6 (95%CI: 1.16–2.2; P = 0.05) times higher for subjects in the highest quartile, 1.57 times higher for subjects in the third quartile (95%CI: 1.18–2.09; P = 0.02) and 1.44 times higher for subjects in the second quartile (95%CI: 1.11–1.88; P = 0.07).

### Receiver-operating characteristic curve analysis of the CVAI and other obesity indices

**Figure 2:**
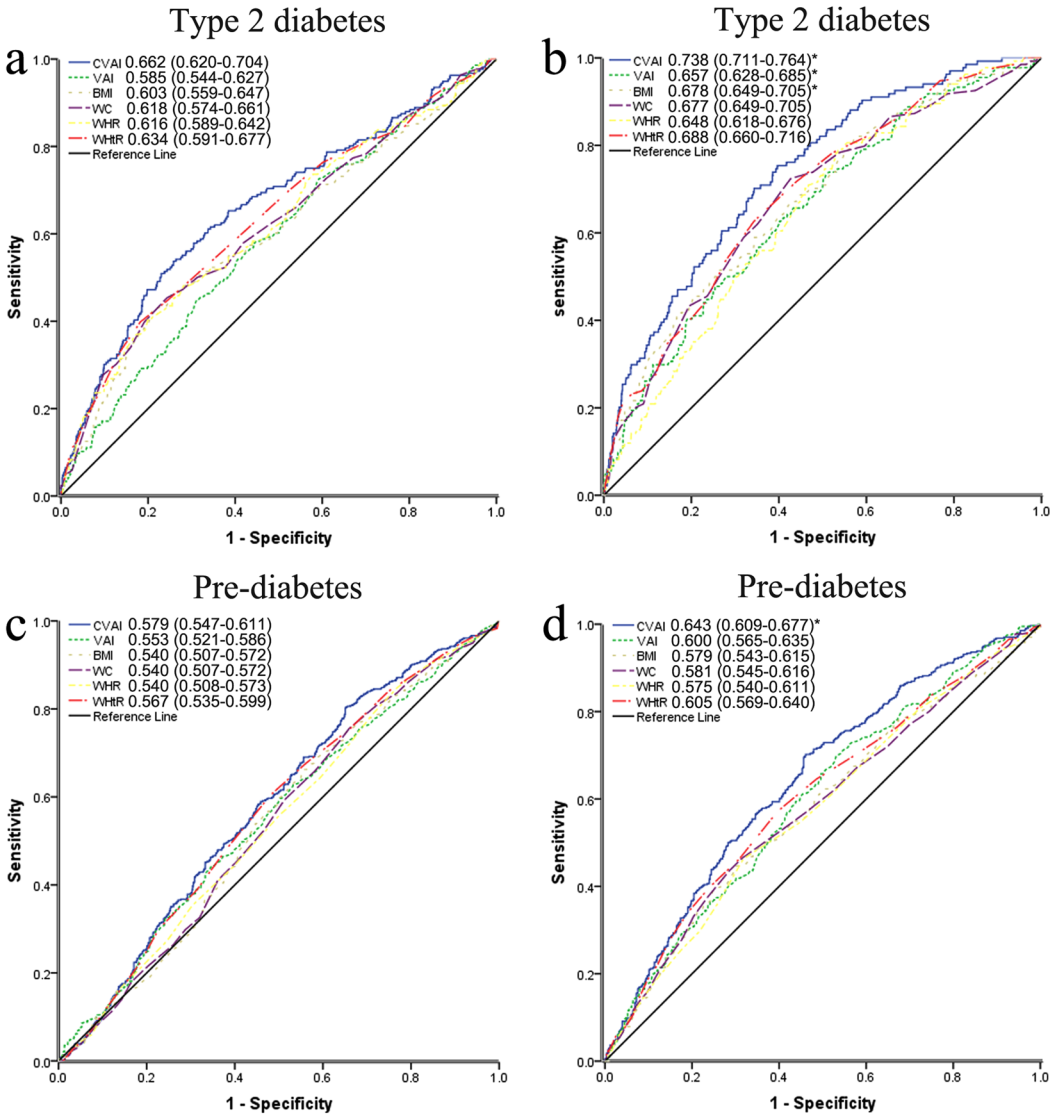
The ROC curves for the CVAI, VAI, BMI, WC, WHR and WHtR for men (**a**,**c**) and women (**b**,**d**) to diagnose type 2 diabetes and pre-diabetes. *Significant differences (P < 0.05) of the AUC for each adiposity index from the men were compared with the method of DeLong *et al*. (1988).

Receiver-operating characteristic (ROC) curve analysis was used to compare the predictive value of obesity indices for diabetes and pre-diabetes by gender and to determine the optimal cut-off values for each index. ROC curve analysis indicated that of the six obesity indices tested, the CVAI had the highest diagnostic accuracy for diabetes (0.662 in male and 0.738 in female) and pre-diabetes (0.620 in male and 0.690 in female) for both genders (Fig. 2). The area under the ROC curve (AUC) for the CVAI was approximately 0.7, which indicates that it had a relatively higher predictive discriminatory power. The VAI, BMI, WC, WHR and WHtR exhibited lower accuracy in predicting diabetes and pre-diabetes for both genders. The ROC curve for VAI, BMI, WC, WHR and WHtR were similar (Supplementary Tables S3 and S4). For the prediction of diabetes, the AUCs for the CVAI, VAI and BMI were significantly (P < 0.05) higher in females than in males. Tables 2 and 3 show the optimal cut-off values and corresponding sensitivity and specificity of each index for identifying diabetes and pre-diabetes by gender. We found that the CVAI had the greatest Youden index values for identifying diabetes and pre-diabetes both in men (0.28/0.15) and women (0.36/0.24). The optimal CVAI cut-off for predicting diabetes was 116.65 in males and 89.65 in females, and the optimal CVAI cut-off for predicting pre-diabetes were 75.5 in males and 71.65 in females. For all obesity indices, the Youden index value was much lower for the identification of pre-diabetes than for diabetes.

**Table 2:** Sensitivity, specificity and Youden index using sex-specific cut-off points for various visceral obesity indices to predict diabetes over 5 years.

	Male				Female			
	Cut-off	Sens (%)	Spec (%)	Youden index	Cut-off	Sens (%)	Spec (%)	Youden index
CVAI	116.65	50.93	76.96	0.28	89.65	70.15	65.66	0.36
VAI	2.04	44.91	68.47	0.13	2.05	62.69	59.83	0.23
BMI	26.08	42.13	79.20	0.21	24.92	45.52	79.71	0.25
WC	89.00	45.40	75.70	0.21	77.00	72.40	57.30	0.30
WHR	0.91	43.10	75.80	0.19	0.82	79.10	46.51	0.26
WHtR	0.54	39.80	81.80	0.22	0.50	62.69	65.66	0.28

**Table 3:** Sensitivity, specificity and Youden index using sex-specific cut-off points for various visceral obesity indices to predict pre-diabetes over 5 years.

	Male				Female			
	Cut-off	Sens (%)	Spec (%)	Youden index	Cut-off	Sens (%)	Spec (%)	Youden index
CVAI	75.30	80.41	34.78	0.15	71.61	70.20	53.92	0.24
VAI	1.88	44.03	65.52	0.10	1.53	69.95	46.85	0.17
BMI	23.50	65.86	44.83	0.11	23.73	44.58	70.50	0.15
WC	80.00	75.56	34.03	0.10	79.00	43.60	71.43	0.15
WHR	0.84	84.89	23.09	0.08	0.84	46.31	68.20	0.15
WHtR	0.50	60.45	51.87	0.12	0.49	57.14	60.37	0.18

### Prediction of diabetes using obesity indices in subgroups

**Figure 3:**
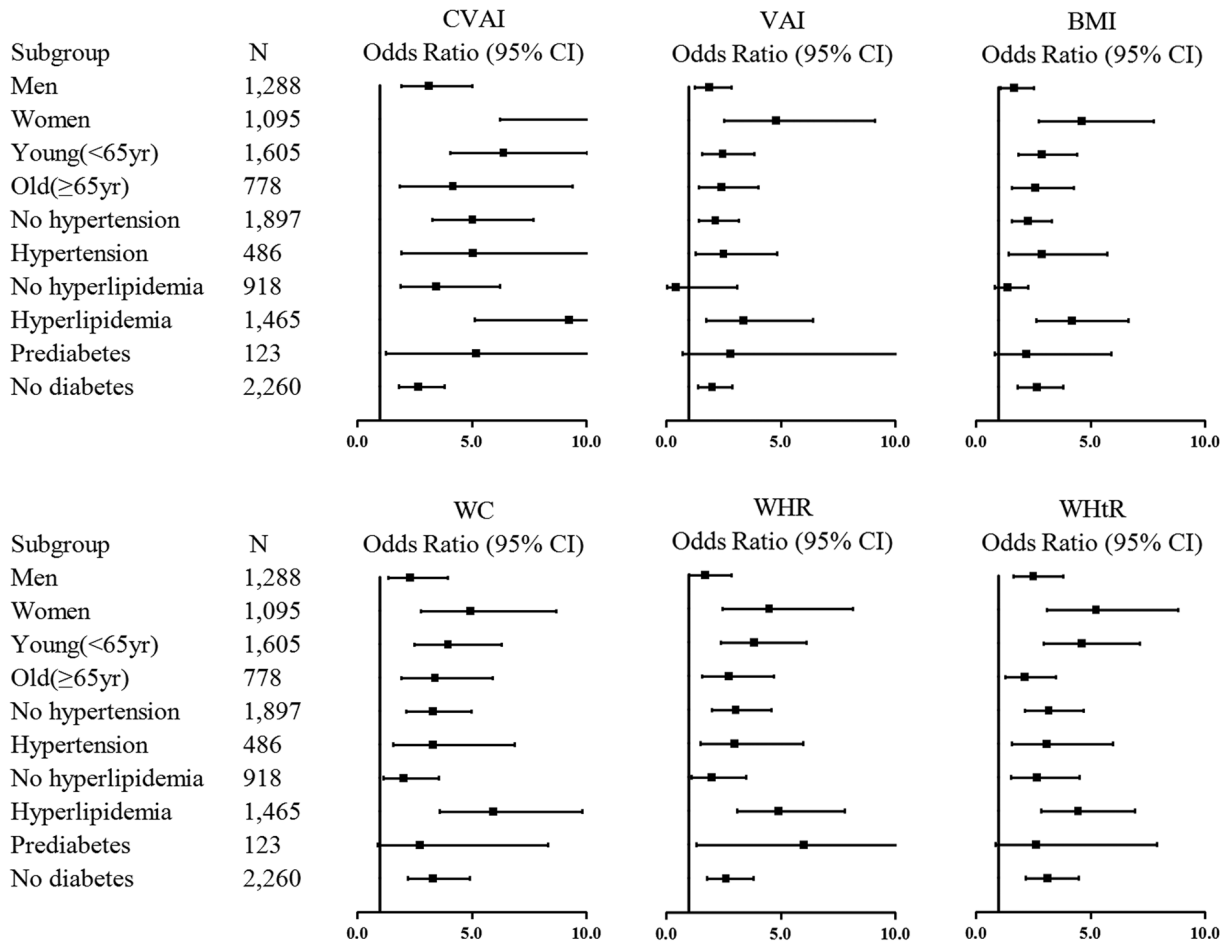
Odds ratio (OR) and 95% confidence intervals (CI) of 5-year incident diabetes for the CVAI, VAI, BMI, WC, WHR and WHtR in various subgroups. Hypertension was defined as systolic/diastolic blood pressure ≥140/90 mmHg, self-report history, or currently taking medications. Hyperlipidemia was defined as TG ≥ 1.7 mmol/L and/or HDL-C < 1.03 mmol/L (men) and HDL-C < 1.29 mmol/L (women). Pre-diabetes was defined as 7.0 mmol/L > FPG ≥ 6.1 mmol/L, 11.1 mmol/L > 2hPG ≥ 7.8 mmol/L, without a history of diabetes previously as well as a current anti-diabetes medication use.

Figure 3 shows the crude OR for the development of diabetes based on subgroup analyses of the top quartile of adiposity indices compared to the first quartile. The correlation between the CVAI and the risk of diabetes was the strongest of all six indices in males and females, all ages and any metabolic health (hypertension, hyperlipidemia and dysglycemia) status. All six obesity indices showed strong relationship with the risk of diabetes in the female subgroup. However, the relationship between diabetes and the WHtR and WC disappeared in the pre-diabetes group, and the relationship between diabetes and the VAI and BMI disappeared in the non-hyperlipidemia and pre-diabetes subgroups.

## Discussion

Our study suggests that the CVAI is superior to the VAI, BMI, WC, WHR and WHtR in predicting diabetes and pre-diabetes in Chinese adults. The incidence of diabetes and pre-diabetes increased with CVAI quartile independent of several well-known risk factors for dysglycemia. The correlation between the CVAI and the risk of diabetes was strong in men and women, old and young, and those with or without hypertension, hyperlipidemia or dysglycemia. Moreover, ROC curve analysis confirmed that of all six indices tested, the CVAI was the best predictor of diabetes and pre-diabetes in both genders.

Visceral obesity is a major risk factor for diabetes^13^; therefore, it is crucial to identify visceral adiposity. MRI and CT provide the most accurate measurement of body fat; however, they are time-consuming and costly. Therefore, surrogate indices, including anthropometric indices (BMI, WC, WHR and WHtR) are widely used. Numerous studies have attempted to determine the optimal anthropometric index to identify metabolic syndrome, cardiovascular disease and other obesity-associated disease^14–16^. A systematic review and meta-analysis showed that the WHtR was a better screening tool than the WC and BMI for detecting cardiometabolic risk factors in males and females^17^. A study conducted in a Taiwanese population suggested that the WHR was the best predictor of the risk of type 2 diabetes among four anthropometric indices^18^. Several studies have concluded that the WHR and WHtR are more effective for predicting metabolic disease than classic anthropometric indices (BMI and WC)^19^, possibly because the WHR and WHtR offer more information than classic anthropometric indices^20^. However, a common shortcoming of all anthropometric indices is their inability to distinguish between peripheral fat and abdominal fat^21^. The VAI, which is based on anthropometric and metabolic measures, was proposed as an indicator of visceral fat^11^. A prospective cohort study conducted in China showed that the VAI is a better surrogate index than single anthropometric indices^22^, although we did not observe a benefit of the VAI in this study. Consistent with other studies^23–25^, we found that the predictive power of different indices were similar. The VAI did not improve the prediction of metabolic syndrome, possibly due to the sample size and differences in the districts of participants.

Recently, the CVAI, a new indicator of visceral adiposity, was developed. A cross-sectional study in China indicated that this indicator is more reliable than the VAI and anthropometric indices for evaluating metabolic risk^12^. Our study extends these studies and, for the first time, evaluates the predictive power of the CVAI for future diabetes and pre-diabetes. Our study showed that the CVAI has many advantages as a predictor of diabetes and pre-diabetes. First, the CVAI was more accurate for predicting diabetes and pre-diabetes than other adiposity indices. The relationship between the CVAI and dysglycemia was the strongest identified in subgroup analyses. In addition, the CVAI showed the highest discriminatory power for dysglycemia in males and females. Second, the CVAI includes many well-known diabetes risk factors, such as a general obesity index (BMI) and a central obesity index (WC). Third, the CVAI was less influenced by body size. The correlation coefficients between the CVAI and height and weight were 0.121 and 0.624, respectively (Supplementary Table S5). Despite its many advantages as an independent predictor of diabetes and pre-diabetes, the predictive value of the CVAI was not as strong as expected. To enhance its value as a predictive tool for diabetes, other risk factors (family history, HC, etc.) may need to be considered.

The main strength of this study is the 5-year prospective design and the large number of subjects. Our data, which included many potential risk factors for diabetes, were relatively comprehensive. In addition, new-onset, baseline diabetes and pre-diabetes were confirmed by self-reported medication use and oral glucose tolerance tests (OGTT). Our study also has some limitations. Most of our study population was older, with a relatively high prevalence of dysglycemia. Our study population lived in China, and further validation may be necessary to generalize our findings to other ethnic groups.

In conclusion, the CVAI was an effective predictor of diabetes and pre-diabetes in Chinese adults. A higher CVAI was associated with a relatively higher risk of dysglycemia, and a lower CVAI was associated with a relatively lower risk of dysglycemia. The predictive power of the CVAI was superior to that of the VAI, BMI, WC, WHR and WHtR in both genders. The CVAI may improve the early identification of patients at high-risk for diabetes and pre-diabetes.

## Methods

### Data collection and the study variables

**Figure 4:**
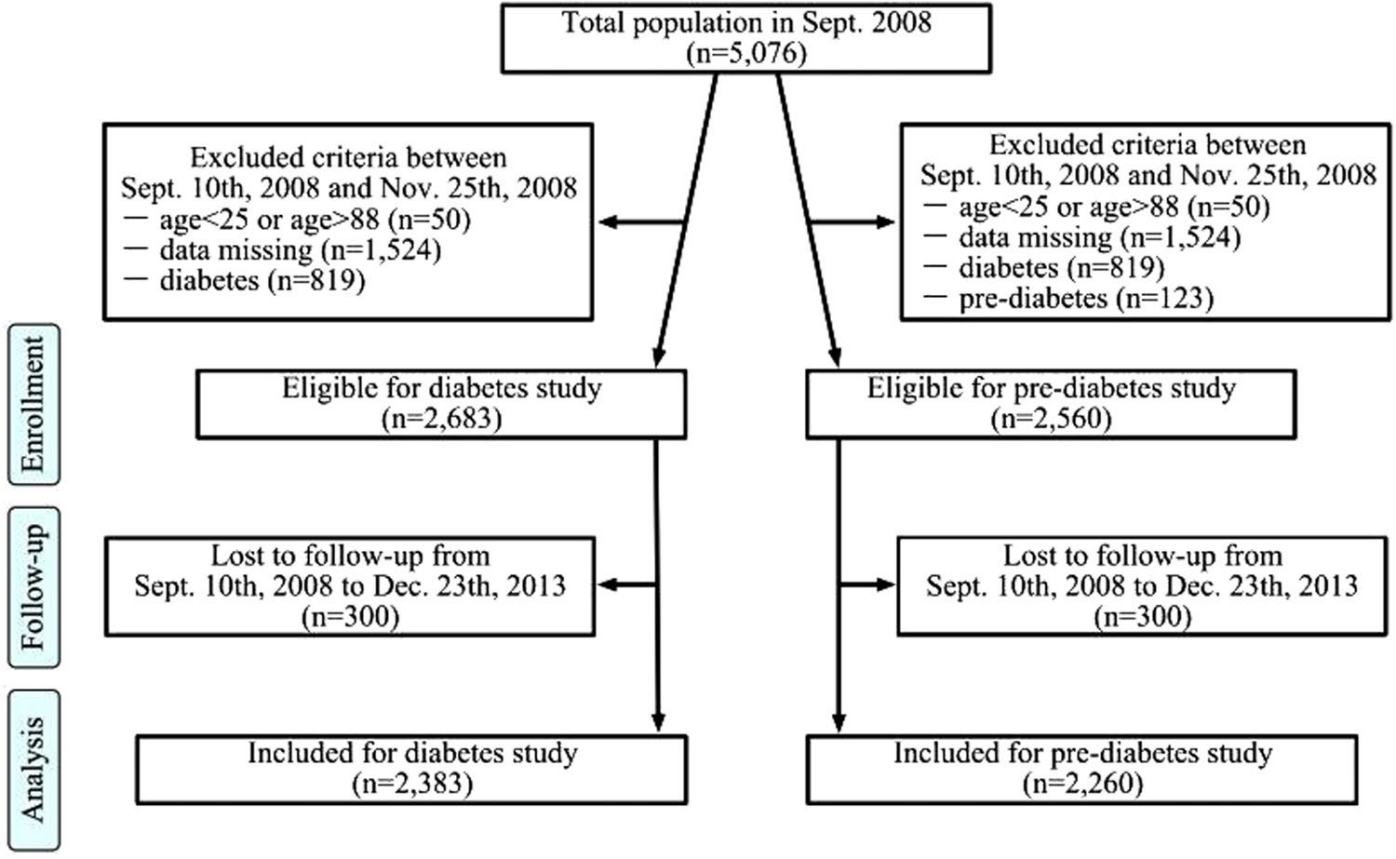
Flow chart of study subjects for examining the incidence of diabetes and pre-diabetes.

This community-based study is a long term program for examining the incidence of metabolic diseases in a large Chinese population. Briefly, the study begun in September 2003, is ongoing, to be continued for at least 20 years. The primary endpoint of this study was death. The present study is a part of it. The present study was designed for and aimed at determining the risk factors and incidence of diabetes, performing regular assessments at 5-year intervals. The primary outcome of the present study is the occurrence of diabetes, the secondary outcome of this study is the occurrence of pre-diabetes. In September 10th, 2008, a total of 5,076 subjects from a community in Chongqing agreed to participate in this follow-up study. The participant selection process is presented in Fig. 4. After exclusion of participants who were aged <25 or >90 years (n = 50) or were missing information on diabetes status (history of diabetes, using glucose-lowering drugs, FPG, or 2hPG), anthropometric measures (BMI, WC, HC, SBP and DBP), or metabolic characteristics (TG, TC, HDL-C and LDL-C) (n = 1,524) and those with diabetes (n = 819), this study consisted of 2,683 subjects. Because this was also a study on the incidence of pre-diabetes, we excluded participants with pre-diabetes (n = 123) at baseline. The final sample for analysis of the incidence of pre-diabetes included 2,260 participants (1,047 males and 950 females). The initial baseline examination took place between September 10th, 2008 and November 25th, 2008. Participants in the study were mainly faculty in a university. This study was approved by the Ethical Committee of the First Affiliated Hospital of Chongqing Medical University and each participant provided written informed consent. All methods described were performed in accordance with the guidelines of the Declaration of Helsinki. The baseline information included data on cigarette smoking (none, former smoker or current smoker), daily alcohol intake (yes or no), anthropometric measurements (height, weight, WC and HC), blood pressure (BP) and laboratory values (TC, TG, HDL-C, LDL-C, FPG and 2hPG). Weight was measured with participants standing and wearing light clothes, and height was measured while participants were barefoot. WC and HC were measured in a standing position with a tapeline between the lowest rib and the iliac crest and at the level of the greater trochanters, respectively. BP was measured with standard techniques using mercury manometers after participants had rested for 10 min in a sitting position. Participants completed detailed self-report questionnaires on cigarette smoking, daily alcohol intake, medical history and medication. All the anthropometric measurements and self-reported questionnaires were obtained by well-trained physicians. BMI was calculated as weight (in kilograms) divided by the square of height (in meters). The WHR was calculated as WC divided by HC. The WHtR was calculated as WC divided by height (in centimeters). Blood samples were drawn from participants in the morning during a fasting state and 2 hours after a 75 g OGTT. OGTT was administrated at both the baseline and follow-up examination for diagnosis of diabetes and pre-diabetes. Laboratory examination data including FPG, 2hPG, TC, TG, HDL-C and LDL-C levels were determined using the hexokinase method. Diabetes was defined as FPG ≥ 7.0 mmol/L, 2hPG ≥ 11.1 mmol/L, self-reported history, or current use of diabetes medication^26^. Pre-diabetes was defined as 7.0 mmol/L > FPG ≥ 6.1 mmol/L, 11.1 mmol/L > 2hPG ≥ 7.8 mmol/L, without a history of diabetes or current use of diabetes medication^27^. Overweight was defined as BMI = 24.0–27.9 kg/m2, and obese was defined as BMI ≥ 28 kg/m2^28^. VAI was calculated as following (both TG and HDL-C were expressed in mmol/L):

$$\begin{array}{c}{\rm{Males}}:{\rm{VAI}}=({\rm{WC}}/39.68+(1.88\times {\rm{BMI}}))\times ({\rm{TG}}/1.03)\times (1.31/{\rm{HDL}}\,-\,{\rm{C}});\\ {\rm{Females}}:{\rm{VAI}}=({\rm{WC}}/36.58+(1.89\times {\rm{BMI}}))\times ({\rm{TG}}/0.81)\times (1.52/{\rm{HDL}}\,-\,{\rm{C}})\end{array}$$

CVAI was calculated by the following formula:

$$\begin{array}{rcl}{\rm{Males}}:{\rm{CVAI}} & = & -267.93+0.68\times {\rm{age}}+0.03\times {\rm{BMI}}+4.00\times \mathrm{WC}(\mathrm{cm})\\  &  & +22.0\times \,\mathrm{Log}10{\rm{TG}}-16.32\times {\rm{HDL}}\,-\,{\rm{C}};\\ {\rm{Females}}:{\rm{CVAI}} & = & -187.32+1.71\times {\rm{age}}+4.23\times {\rm{BMI}}+1.12\times \mathrm{WC}(\mathrm{cm})\\  &  & +39.76\times \,\mathrm{Log}10{\rm{TG}}-11.66\times {\rm{HDL}}\,-\,{\rm{C}}.\end{array}$$

Between October 5th, 2013 and December 23th, 2013, a follow-up examination was carried out. Of the initial cohort, 160 persons had died and 140 had left the university before October 5th, 2013. The remaining 2,383 subjects (1,288 males and 1,095 females) completed the follow-up and were involved in the present analysis. The contents of follow-up examinations were similar to those conducted at baseline. The onset of diabetes was defined as FPG ≥ 7.0 mmol/L, 2hPG ≥ 11.1 mmol/L, self-reported history (from the beginning to the end of this study), or current use of diabetes medication. Pre-diabetes was defined as 7.0 mmol/L > FPG ≥ 6.1 mmol/L, 11.1 mmol/L > 2hPG ≥ 7.8 mmol/L, without a history of diabetes or current use of diabetes medication. Data collected were double double lose form and directly stored in computers as database software- computer assisted system.

### Statistical analysis

All statistical analyses were performed with SPSS version19.0 (SPSS, United States). The Kolmogorov-Smirnov test was used to verify the normal distribution of data. Normal variables were expressed as mean ± standard deviation, while skewed variables expressed as median ± inter-quartile. One-way analysis of variance (ANOVA) was used to compare differences in measurement variables by different CVAI quartiles. Categorical variables were expressed as percentages, and compared by Chi-square test. Logistic regression analysis was used to estimate the associations of each obesity indices with the incidence of diabetes and pre-diabetes. We fitted three models: (a) crude; (b) controlling for age and gender; (c) additionally adjusted for baseline TG, TC, LDL-C, SBP and DBP; We plotted a ROC curve to compare the diagnostic accuracy of the six obesity indices and to find the optimal cut-off values of each index. Differences between AUC were tested with Z statistic. A two-tailed P < 0.05 was accepted as statistical significance in the full text.

### Data Availability

The datasets analyzed during the current study are available from the corresponding author on reasonable request.

## Electronic supplementary material

Supplemantary Information
