# Supplementary material for: A Novel Visceral Adiposity Index for Prediction of Type 2 Diabetes and Pre-diabetes in Chinese adults: A 5-year prospective study
Source: Sci Rep. 2017 Oct 23;7:13784. doi: 10.1038/s41598-017-14251-w (PMC5653832; doi:10.1038/s41598-017-14251-w)
Supplement: Supplementary file 1 — Supplemantary Information [file 41598_2017_14251_MOESM1_ESM.doc]

A Novel Visceral Adiposity Index for Prediction of Type 2 Diabetes and Pre-diabetes in Chinese adults: A 5-year prospective study

Jinshan Wu^1^, Lilin Gong^1^, Qifu Li^1^, Jinbo Hu^1^, Shuping Zhang^1^, Yue Wang^1^, Huang Zhou^1^, Shuming Yang^1^, Zhihong Wang^1*^

^1^The First Affiliated Hospital of Chongqing Medical University, Department of Endocrinology, Chongqing, 400016, China.

**Supplementary Table 1:** Baseline characteristics of the males and females.

|  | Males  (n=1,288) | Females  (n=1,095) | *P*-value |
| --- | --- | --- | --- |
| Age (years)** | 58.50 ± 12.99 | 55.09 ± 11.51 | <0.001 |
| Height (cm)** | 166.08 ± 6.11 | 155.83 ± 5.85 | <0.001 |
| Weight (kg)** | 66.96 ± 9.08 | 56.17 ± 7.42 | <0.001 |
| WC (cm)** | 84.76 ± 7.62 | 77.31 ± 7.73 | <0.001 |
| HC (cm)** | 95.43 ± 5.42 | 92.69 ± 5.75 | <0.001 |
| BMI ** | 24.24 ± 2.73 | 23.13 ± 2.79 | <0.001 |
| WHR** | 0.89 ± 0.05 | 0.83 ± 0.05 | <0.001 |
| WHtR** | 0.51 ± 0.04 | 0.50 ± 0.05 | 0.626 |
| CVAI** | 96.66 ± 33.66 | 80.51 ± 36.26 | <0.001 |
| VAI** | 1.87 ± 1.17 | 2.27 ± 1.61 | <0.001 |
| SBP (mmHg)** | 120 (110,130) | 114 (104,126) | <0.001 |
| DBP (mmHg)** | 80 (70,80) | 70 (70,80) | <0.001 |
| FPG (mmol/L)** | 4.23 ± 0.60 | 4.20 ± 0.55 | 0.173 |
| 2hPG (mmol/L)** | 5.31 ± 1.35 | 5.08 ± 1.14 | <0.001 |
| TG (mmol/L)** | 1.52 ± 0.79 | 1.37 ± 0.75 | <0.001 |
| TC (mmol/L)** | 4.58 ± 0.75 | 4.75 ± 0.79 | <0.001 |
| HDL-C (mmol/L)** | 1.09 ± 0.20 | 1.20 ± 0.27 | <0.001 |
| LDL-C (mmol/L)** | 2.90 ± 0.61 | 2.95 ± 0.68 | 0.094 |
| Overweight, n (%)* | 570 (44.3) | 303 (27.7) | <0.001 |
| Obese, n (%)* | 115 (8.9) | 65 (5.9) | 0.006 |
| Smoking (Current and  Former smokers), n (%)* | 494 (38.4) | 11 (0.01) | <0.001 |
| Drinking, n (%)* | 211 (16.4) | 9 (0.008) | <0.001 |

*Percentage and *P*-value by the chi-square test. **Mean (standard deviation) and *P*-value by the unpaired t-test or non-parametric tests.

Abbreviations: WC, waist circumference; HC, hip circumference; WHR, waist-to-hip ratio; WHtR, waist-to-height ratio; CVAI, Chinese Visceral Adiposity Index; VAI, Visceral Adiposity Index; SBP, systolic blood pressure; DBP, diastolic blood pressure; FPG, fasting plasma glucose; 2hPG, 2 hours postload plasma Glucose; TG, triglyceride; TC, total cholesterol; HDL-C, high-density lipoprotein cholesterol; LDL-C, low-density lipoprotein cholesterol.

**Supplementary Table 2**: Incident cases of diabetes and pre-diabetes after 5-year follow up according to the baseline quartiles of CVAI.

|  | Baseline CVAI | | | | | | | | | | |  |
| --- | --- | --- | --- | --- | --- | --- | --- | --- | --- | --- | --- | --- |
|  | 1st | 2nd | | | | 3rd | | 4th | | *P* value | |  |
|  | < 63.84 | 63.84 - 88.74 | | | | 88.75 - 113.88 | | > 113.88 | |  | |  |
|  | (n=596) | (n=596) | | | | (n=596) | | (n=595) | |  | |  |
| Incident diabetes, n (%) | 37 (6.2) | 59 (9.9) | | | | 81 (13.6) | | 173 (29.8) | | < 0.001 | |  |
|  | 1st | 2nd | | | | 3rd | | 4th | | *P* value | |  |
|  | <62.72 | 62.72-87.23 | | | | 87.23-111.75 | | >111.75 | |  | | |
|  | (n=565) | | (n=566) | | (n=565) | | (n=564) | |  | |  | |
| Incident Pre-diabetes, n (%) | 152 (26.9) | | | 227 (40.1) | 264 (46.7) | | 299 (53.0) | | < 0.001 | |  | |

**Supplementary Table 3**: *P* values for pairwise comparison of ROC curves for different adiposity indices in men and women (diabetes).

|  | Men | | | | | | |  | | Women | | | | | | |  |
| --- | --- | --- | --- | --- | --- | --- | --- | --- | --- | --- | --- | --- | --- | --- | --- | --- | --- |
|  | | CVAI | VAI | BMI | WC | WHtR | WHR | |  | | CVAI | VAI | BMI | WC | WHtR | WHR | |
| CVAI | | 1.0000 | 0.0001 | 0.0001 | <0.00011 | 0.0032 | 0.0021 | |  | | 1.0000 | 0.0001 | 0.0013 | 0.0006 | 0.0029 | 0.0001 | |
| VAI | |  | 1.0000 | 0.47370.3860 | 0.1508 | 0.0377 | 0.2185 | |  | |  | 1.0000 | 0.5005 | 0.4754 | 0.2701 | 0.7465 | |
| BMI | |  |  | 1.0000 | 0.2552 | 0.0275 | 0.5404 | |  | |  |  | 1.0000 | 0.9791 | 0.4686 | 0.2503 | |
| WC | |  |  |  | 1.0000 | 0.0585 | 0.8879 | |  | |  |  |  | 1.0000 | 0.2916 | 0.1284 | |
| WHtR | |  |  |  |  | 1.0000 | 0.21371 | |  | |  |  |  |  | 1.0000 | 0.0276 | |

**Supplementary Table 4**: *P* values for pairwise comparison of ROC curves for different adiposity indices in men and women (pre-diabetes).

|  | Men | | | | | |  | Women | | | | | |
| --- | --- | --- | --- | --- | --- | --- | --- | --- | --- | --- | --- | --- | --- |
|  | CVAI | VAI | BMI | WC | WHtR | WHR |  | CVAI | VAI | BMI | WC | WHtR | WHR |
| CVAI | 1.0000 | 0.1044 | 0.0013 | <0.0001 | 0.1187 | 0.0008 |  | 1.0000 | 0.0041 | <0.0001 | <0.0001 | 0.0018 | 0.0001 |
| VAI |  | 1.0000 | 0.47110.3860 | 0.4583 | 0.4485 | 0.5080 |  |  | 1.0000 | 0.3180 | 0.3330 | 0.8236 | 0.2442 |
| BMI |  |  | 1.0000 | 0.9765 | 0.0169 | 0.9643 |  |  |  | 1.0000 | 0.8898 | 0.0348 | 0.8449 |
| WC |  |  |  | 1.0000 | 0.0002 | 0.9700 |  |  |  |  | 1.0000 | 0.0005 | 0.6367 |
| WHtR |  |  |  |  | 1.0000 | 0.0141 |  |  |  |  |  | 1.0000 | 0.0149 |

**Supplementary Table 5**: Correlation coefficient between adiposity indices and the body size (height and weight).

|  | Height | Weight |
| --- | --- | --- |
| CVAI | 0.137^**^ | 0.614^**^ |
| VAI | -0.046^**^ | 0.198^**^ |
| BMI | 0.115^**^ | 0.784^**^ |
| WC | 0.416^**^ | 0.815^**^ |
| WHtR | -0.024^**^ | 0.551^*^ |
| WHR | 0.325^**^ | 0.574^**^ |

^**^*P*<0.001, ^*^ *P*<0.05.
